# The Transactions of the Provincial Medical and Surgical Association. Vol. XIV

**Published:** 1846-04

**Authors:** 


### The Transactions of the Provincial Medical and Surgical Association. Vol. XIV. 8vo. 1846.

This volume contains but little that calls for notice. Eighty pages out of the 300 are occupied with a list of the members, &c.; and no fewer than 142 are devoted to the Retrospective Addresses on Medicine and Surgery, by Dr. Charlton of Newcastle, and Mr. Teale of Leeds. Would it not be better that these addresses should now be discontinued, or at least cease to be published, seeing that there is more than one periodical which professes to give yearly or half-yearly retrospects on different branches of medical science? With the talent and large experience of the members of the Association, the Council could find no difficulty in procuring a sufficient number of original papers for their Transactions, after the manner of the Medico-Chirurgical and other Societies. Besides the two Addresses, there is a long and elaborate report of the Reading Dispensary, from 1841 to 1844, by Dr. Cowan; and a minute statistical Report of the Surgical in-patients of the Berkshire Hospital, from its establishment in 1839, by Mr. May. The remaining articles are one on Grinder's Asthma by Dr. Favell of Sheffield, and an interesting case of Inverted Displacement of the Bladder, by Mr. Crosse: these we shall briefly notice.

I. The main design of Dr. Favell is to shew that all the symptoms of "grinder's asthma" (a very inappropriate appellation), as well as the pathological changes induced by it in the respiratory organs, are due to congestion or inflammation of the substance of the lungs; in some cases, giving rise to formation of tubercle, and in others, occasioning pulmonary degeneration without tubercular deposit. The primary and essential microscopic appearances are inflammation and sometimes ulceration of the mucous lining of the air-passages; dispersed masses of the pulmonary parenchyma in a state of induration, the result of a slow inflammatory action; small dark "currant-like bodies" (the dilated extremities of veins, containing some of the solid constituents of the blood) on the surface and in the substance of the lungs; and lastly, tuberculous deposits. In addition to these changes, there are often extensive adhesions of the pleura, dilatation of the bronchi, emphysema of the lungs, &c. Dr. F., as well as Dr. Knight, is of opinion that the fine particles of the grinding-stones do more injury to the workmen than the particles of the iron; being led to this conclusion by the circumstance that, in post-mortem examinations, it is common to find depositions of calcareous matter in the bronchial glands and tubes, whereas "there is no instance on record of any particle of iron or steel having ever been detected" in these parts. Moreover, it is well known that stone-cutters and leather-dressers are almost as liable to pulmonary affections as grinders. Dr. Hodgkin is therefore mistaken, when he attributes the pernicious effects of the occupation of the latter to the atmosphere of their workshops being loaded with the fine metallic particles.*

The tendency to pulmonic disease in grinders, from the direct inhalation of the irritating dust to which they are exposed, is greatly aggravated by the reckless dissipated character of their lives; by their unnecessary exposure to the vicissitudes of the weather, in passing as they often do from heated workshops into the open air, even in Winter; and by the stooping, leaning-forward attitude of their bodies, while at work, so embarassing to the easy play of the lungs.

With respect to prophylactic treatment, much may be done by having the workshops properly ventilated and provided with a flue to carry off the floating dust to the outside of the building, as well as with a suitable arrangement of a series of magnets to attract the particles of steel, also by improving, at the same time, the social habits of the men, in reference to diet, clothing, and general hygiene. "If the moral status of the grinder were elevated, his habits of intemperance would be subdued, the whole system would be better nourished, and, consequently, there would be less liability to the formation of unhealthy deposits on the occurrence of incidental pulmonary inflammation."

II. Mr. Crosse's patient was a child between two and three years of age. There was a fleshy-looking tumour projecting out between the labia pudendi: it was considered a vascular excrescence by the medical attendant; but, before he proceeded to put a ligature round it, he fortunately asked Mr. C. to see it. This gentlemen was puzzled at first what to make of the case; but he saw quite enough to dissuade the intended operation. The following is the description of the appearances:—"Towards the posterior part of the tumour, and on its sacral aspect, there was an aperture, which was conjectured to be the entrance into the displaced urethra. A very small female catheter easily entered this aperture, and passed along a channel a little to the left side of the median line: urine distilled in drops through the catheter, but there was not a gush, although the instrument had entered so far that we concluded it must have reached the cavity of the bladder. Besides what thus oozed through the catheter, slightly tinged with blood, there was an oozing of urine from another source, which was not explained until a second and more strict examination, instituted a few days afterwards.

He then "found, concealed in a fold of the tumour, and near to the posterior junction of the labia, two orifices not far asunder, from which the urine oozed, and which were evidently the vesical terminations of the ureters. On pressing the tumour firmly, as if to reduce it like a hernia, I found it yield and pass gradually behind the symphysis pubis, and within the labia; and under a continuance of the taxis it all retired, leaving the external parts in their proper shape and position. A passage remained, through which the tumour on retiring had taken its course, which was actually the dilated urethra, into which I *could* and *did* introduce my little finger, until it fairly entered the cavity of the replaced bladder; for it now became clearly demonstrated, that the vascular red tumour, externally presenting itself as first described, was the urinary bladder in its entire thickness, including its mucous, muscular, and peritoneal coats, prolapsed

* Lectures on the Morbid Anatomy of Serous and Mucous Membranes. Vol 2.

through the dilated urethra, and at the same time inverted or turned inside out."

The previous history of this case was imperfect. All that could be made out was, that the tumour had existed for a considerable time, and been always attended with a *stillicidium urinæ*. Mr. Crosse has lately learned that his patient is still living, and is now a healthy young woman, 18 years of age; but that unfortunately the incontinence of urine continues, although there has never been any relapse of the vesical inversion.

Dr. Murphy has related, in the Medical Gazette for 1833, a case very similar to the preceding one: it occurred in a female child four years old. At first, it was mistaken for a *prolapsus ani:* a more attentive examination of the parts disclosed the real nature of the displacement.

Mr. Crosse remarks that "when, from difficult labour, the bladder and contiguous part of the vagina sloughs away, creating a free communication between these two cavities, a real inversion of the bladder may take place through this abnormal aperture, of which I have known several instances; and in one, the inversion prolapsed so far as to escape at the external labia, and presented on its surface the terminations of the ureters, from which the urine was observed to dribble continually; when the injury is to this extent, the afflicting symptoms can only be palliated, and have not, to my knowledge, admitted of cure."

